# Structural Analysis, Molecular Modelling and Preliminary Competition Binding Studies of AM-DAN as a NMDA Receptor PCP-Site Fluorescent Ligand

**DOI:** 10.3390/molecules24224092

**Published:** 2019-11-13

**Authors:** Sethu Ndzibongwana, Samukelo Ngobese, Ahmad Sayed, Ciniso Shongwe, Simon White-Phillips, Jacques Joubert

**Affiliations:** Pharmaceutical Chemistry, School of Pharmacy, University of the Western Cape, Private Bag X17, Bellville 7535, South Africa; 3677287@myuwc.ac.za (S.N.); 3677295@myuwc.ac.za (S.N.); 3656144@myuwc.ac.za (A.S.); 3640964@myuwc.ac.za (C.S.); 3509274@myuwc.ac.za (S.W.-P.)

**Keywords:** NMDAR, amantadine, dansyl, fluorescent ligand, energy minima, molecular modelling, fluorescent bioassay

## Abstract

Excitotoxicity related to the dysfunction of the *N*-methyl-d-aspartate receptor (NMDAR) has been indicated to play an integral role in the pathophysiology of multiple disease states, including neurodegenerative disorders such as Parkinson’s disease. There is a notable gap in the market for novel NMDAR antagonists, however current methods to analyse potential antagonists rely on indirect measurements of calcium flux and hazardous radioligand binding assays. Recently, a fluorescent NMDAR ligand, *N*-adamantan-1-yl-dimethylamino-1-naphthalenesulfonic acid, known as AM-DAN was developed by our group. Additional studies on this ligand is necessary to evaluate its potential as a biological tool in NMDAR research. Therefore, this study was aimed at conducting structural analyses, fluorescence experiments, high-accuracy NMDAR molecular modelling and NMDAR phencyclidine (PCP) site competition binding studies using AM-DAN. Results revealed that AM-DAN has appropriate structural properties, significant fluorescent ability in various solvents and is able to bind selectively and compete for the PCP-binding site of the NMDAR. Therefore, AM-DAN holds promise as a novel fluorescent ligand to measure the affinity of prospective drugs binding at the NMDAR PCP-site and may circumvent the use of radioligands.

## 1. Introduction

The dysfunction of the *N*-methyl-d-aspartate receptor (NMDAR) plays an integral role in the pathophysiology of multiple disease states, including ischemia and neurodegenerative disorders such as Parkinson’s- and Alzheimer’s disease. The significance of this receptor and mechanisms involved in its modulation have long been the focus of drug design studies, especially within neurodegenerative disorders, with the primary aim of decreasing neuronal excitotoxicity [1,2,3,4]. 

**Figure 1:**
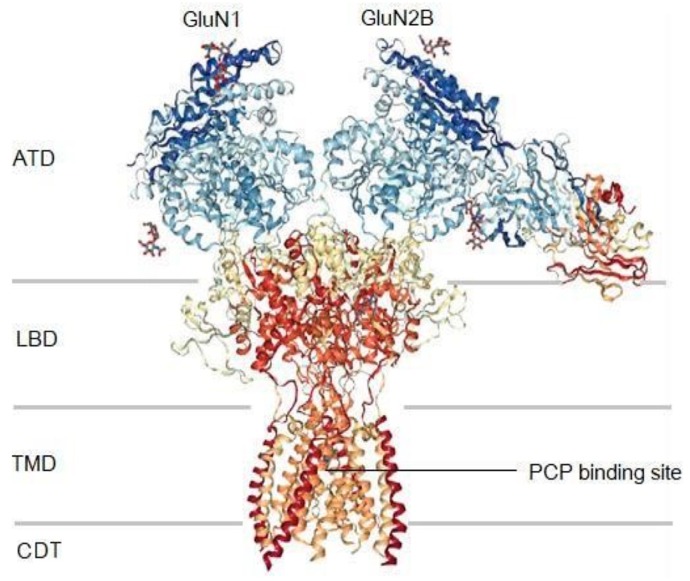
The protein structure of the NMDAR (PDB ID: 5UOW) showing the CDT, TMD, ATD and LBD regions of the GluN1 and GluN2B subunits and the PCP binding site [9].

The NMDAR is an ionotropic glutamate receptor involved in neuroplasticity and cognitive function including memory and learning [1,3,5]. Studies have demonstrated that activation of the NMDAR results in the influx of Ca^2+^ and Na^+^ via the co-agonist binding of d-serine/glycine and glutamate to the NMDAR GluN1 and GluN2 subunits, respectively (Figure 1) [1,3,5]. Each subunit can be divided into three regions; (i) intracellular carboxy-terminal domain (CTD), (ii) transmembrane domain (TMD), and (iii) two extracellular domains, namely the ligand binding domain (LBD) and the amino-terminal domain (ATD) (Figure 1) [1]. Ca^2+^ flux is a necessary mechanism involved in neuroplasticity. However, excessive influx of Ca^2+^ due to NMDAR hyperactivity and hyperglutamatergic transmission is known to invoke excitotoxicity and neurodegeneration [1,5]. Thus, the NMDAR and specifically the inhibition thereof, has become a key target in combating neurodegenerative disorders. 

The NMDAR offers a broad degree of modulation at various sites on the receptor. Some of the most important sites for modulation are the glycine- and glutamate binding sites within the LBD and the PCP-binding site within the TMD (Figure 1). Therefore, modulation of cellular activity, such as Ca^2+^ flux, is possible through the binding of ligands to these sites [1,3,5,6,7,8,9]. Amantadine, an FDA approved drug, and MK-801 are two such examples of non-competitive NMDAR antagonists producing neuroprotective effects. Studies have identified their mechanism of action to be associated with the phencyclidine (PCP) binding site within the TMD of the NMDAR channel [6,7] (Figure 1). The PCP-site is therefore of interest as a target for blocking Ca^2+^ ion flux through the receptor channel and has been considered in multiple drug affinity studies involving drug inhibitors of this binding site [2,6,7,8]. 

Methods currently used to analyse and quantify drug binding at the PCP-site involves radioligand binding studies and indirect measurements of Ca^2+^ flux [2,10,11,12,13]. Radioligand binding techniques specifically, have been widely used to determine the involvement of the NMDAR in the pathophysiology of neurodegenerative disorders [10,13]. While radioligand binding techniques are useful in that they provide a great level of sensitivity, they are associated with a number of problems. High running costs, health hazards, waste disposal and a relatively high technical difficulty associated with operating the equipment are all concerns with radioligand analysis [14]. Consequently, it is necessary to consider alternative methods for the determination of drug-PCP binding interactions. Fluorescent binding assays may be considered a suitable alternative as they are efficient in measuring ligand-target interactions and don’t pose most of the health, safety and environmental issues of radioligand assays [15]. Fluorescent binding techniques can also provide information which is not readily available with radioligand binding studies [16,17].

**Figure 2:**
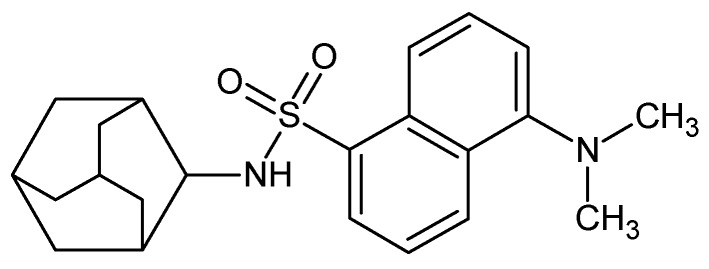
Structure of AM-DAN (*N*-adamantan-1-yl-dimethylamino-1-naphthalenesulfonic acid).

In 2011, Joubert et al., synthesised a series of amantadine-derived compounds, with the aim of determining inhibitory activities of these compounds on Ca^2+^ flux due to NMDAR overstimulation [2]. The study utilised various heterocyclic moieties attached to known NMDAR inhibitors. Among the synthesised compounds was *N*-adamantan-1-yl-dimethylamino-1-naphthalenesulfonic acid (AM-DAN) (Figure 2), a compound composed of a conjugated adamantane and dansylamide moiety, which showed a high degree of NMDAR antagonism. Dansylamides have been used in assay development and other experiments due to their fluorescent nature for which changes in the wavelength of absorbed light may be quantified and recorded by Stokes shifts [2,16,18]. Seeing that AM-DAN should have useful fluorescent properties and have relatively good inhibitory properties for the NMDAR (IC_50_ = 15 µM) compared to amantadine (IC_50_ = 18 µM), it is considered a suitable candidate molecule for the use in the development of a NMDAR fluorescent binding affinity assay [2]. It is also suggested that AM-DAN may bind to the PCP-binding site of the NMDAR because of the inclusion of the adamantane moiety. There is currently a gap in the market for a fluorescent bioassay to directly measure drug-PCP binding and AM-DAN may be a suitable fluorescent ligand to be used in the development of such a bioassay. 

The objectives of this study were therefore to explore the potential of AM-DAN as a fluorescent ligand for bioassay development. To reach this aim the structure of AM-DAN was studied using available X-ray crystallography data [19], energy optimisation experiments, fluorescence studies and molecular orbital analysis. In addition, the NMDAR protein structure (Figure 1: PDB ID: 5UOW [9]) was selected to conduct a series of in silico structure-based molecular modelling experiments. This NMDAR protein was selected because it is complexed with the co-agonists glycine and glutamate, leading to the activation thereof in its open-channel state [1,3,5,9]. Because the channel is activated, ligands are able to move through the channel and enabled the co-complexed ligand, MK-801, to bind to the PCP-site. This protein was therefore deemed appropriate for the docking experiments in order to explore the potential binding of AM-DAN to the PCP-binding site. Furthermore, a NMDAR competition binding study was conducted as a proof-of-concept for the use of AM-DAN as a fluorescent ligand to measure drug-PCP binding.

## 2. Materials and Methods

### 2.1. Synthesis of AM-DAN 

The synthesis procedure and crystallisation of AM-DAN was conducted as previously described by our group. The physical characteristics were in agreement as reported [19,20]. Physical data: ^1^H NMR (400 MHz, CDCl_3_) δ_H_: 8.53–8.51 (dd, *J* = 6.2 and 2.2 Hz, 1H), 8.31–8.30 (dd, *J* = 6.3 and 2.2 Hz, 1H), 8.25–8.23 (dd, *J* = 6.3 and 2.1 Hz, 1H), 7.58–7.50 (m, 2H), 7.19–7.17 (dd, *J* = 6.4 and 2.1 Hz, 1H), 2.90 (s, 6H, N(CH_3_)_2_), 1.95 (s, 3H), 1.74 (d, 6H), 1.59 (m, 6H); ^13^C NMR (100 MHz, CDCl_3_) δ_C_: 151.9, 138.7, 129.9, 129.6, 128.9, 128.0, 123.4, 119.2, 115.0, 55.5, 45.5, 43.0, 35.2, 29.5. HREI-MS: Calculated for C_22_H_28_N_2_O_2_S (M + H^+^); 385.1950, found 385.1944. 

### 2.2. Energy Minima Studies 

Energy minimisation of AM-DAN was calculated using Avogadro 1.2.0 software [21,22]. The crystal structure of AM-DAN was retrieved from the Cambridge Crystallographic Data Centre (CCDC, deposition number: 1949154). A crystal unit within the crystal structure of AM-DAN was selected as the starting structure for the energy minimisations calculations. The structure was imported into the software and exposed to a simulated molecular mechanics force field (MMFF94). Geometry optimisation was set at 9999 steps, steepest decent with a convergence of 10^−7^. This energy minimisation process optimised the AM-DAN molecule into its lowest possible energy state (i.e., removed from the influence of the crystal field and intermolecular interactions). This minimised structure was further used in the molecular orbital- and docking studies.

### 2.3. HOMO and LUMO Calculations 

The MMFF94 (gas phase) energy minimised structure of AM-DAN generated by Avogadro 1.2.0 was opened within the Molecular Operating Environment (MOE) 2018 software suite [23]. The Highest Occupied Molecular Orbital (HOMO) and Lowest Unoccupied Molecular Orbital (LUMO) were then rendered within the software for this optimised structure. 

### 2.4. Fluorescence Studies 

All fluorescent experiments were conducted using a BioTek Synergy (BioTek Instruments, Inc., Winooski, VT, USA, 2013) fluorescent reader. A 1 mM stock solution of AM-DAN in dimethyl sulphoxide (DMSO) was made. To a 96 well black plate, 200 µL of the following solvents were then added to each respective well: DMSO, dimethylformamide (DMF), toluene, tetrahydrofuran (THF) and an aqueous DMEM solution (Dulbeco Modified Eagles Medium, Gibco, Life Technologies Ltd., Paisley., Renfrewshire., UK). The 1 mM AM-DAN stock solution was then transferred into each individual solvent well (2 µL of stock solution per well, final concentration of 10 µM). The wells were covered to prevent evaporation and agitated gently. The samples were then irradiated at an excitation of 340 nm and the emission values were measured. Solid state fluorescence was done by placing the AM-DAN crystals (as prepared in Section 2.1) in a single well, ensuring the bottom of the well was covered with the crystals. The crystals were then irradiated (340 nm) and the emission was measured.

### 2.5. Molecular Modelling 

#### 2.5.1. Blind Docking 

Blind docking was conducted with the PyRx virtual screening tool [24] to ascertain the most probable site for binding of AM-DAN within the NMDAR. The NMDAR protein structure (5UOW [9]) was obtained from the online available Protein Data Bank and prepared as described in Section 2.5.2. A blind docking grid was placed over the entire NMDAR protein structure. The co-complexed ligands (MK-801, glutamate and glycine) were removed from the protein structure before commencement of the docking experiment. The MMFF94 optimised structure of AM-DAN was saved as a pdb file and imported into the PyRx database. Different poses of AM-DAN were computed and compared to determine the potential site of binding within the NMDAR. To validate this docking protocol, the co-complexed ligand, MK-801, was re-docked into the NMDAR using this blind docking protocol. This procedure was repeated three times and the top ranked poses (according to binding energy), in each case, was predicted to bind to the PCP-site with RMSD values of less than 2.0 Å from the position of the co-complexed MK-801. In general, RMSD values smaller than 2.0 Å, indicate that the docking protocol is capable to accurately predict the binding the site of a ligand [25,26]. This protocol was thus deemed to be suitable for the blind docking experiment.

#### 2.5.2. In Silico Structure-based Docking Studies

Site-specific molecular docking of AM-DAN within the PCP-binding site was conducted using the NMDAR protein structure (Protein Data Bank I.D.: 5UOW [9]). The methods used are similar to those originally reported by Joubert et al., 2018 and 2019 [27,28], with the exception of the protein used for the docking experiments. The Molecular Operating Environment (MOE) 2018 software suite [23] was used for docking studies with the following protocol: (i) the enzyme protein structure was checked for missing atoms, bonds, and contacts, (ii) removal of water molecules, 3D protonation and energy minimisation was done with parameters, force field: MMFF94X+solvation, gradient: 0.05, chiral constraint and current geometry. This minimised structure was used as the receptor for docking analysis. (iii) The MMFF94 optimised structure of AM-DAN was saved as a pdb file and imported into the MOE database. (iv) AM-DAN was then docked within the PCP-site by means of the MOE Dock application. The active site was designated based on the proximity of the co-complexed ligand, MK-801, with the help of the MOE Site Finder tool. The docking algorithm which was chosen for these experiments was based on induced fit docking to allow for flexible interactions of AM-DAN with the protein. (v) The best binding pose of AM-DAN was visually examined, and the interactions with the binding pocket residues were analysed. The selected parameters that were used to calculate the score and interaction of the ligand molecule with the NMDAR were as follows: Rescoring function, London dG; Placement, Triangle matcher; Retain, 30; Refinement, Force field; Rescoring 2, London dG. The build in scoring function of MOE, S-score, was used to predict the lowest binding affinity (kcal/mol) of AM-DAN with the NMDAR PCP-site after docking. To validate this docking protocol, the complexed ligand, MK-801, was re-docked into the PCP-binding site. This procedure was repeated three times and the top best ranked pose of MK-801 (according to binding affinity), in each case, exhibited an RMSD value of less than 1.0 Å from the position of the co-complexed MK-801. In general, RMSD values smaller than 2.0 Å, indicate that the docking protocol is capable of accurately predicting the binding orientation of the co-crystallised ligand [25,26]. This protocol was thus deemed to be suitable for the docking of ligands into the PCP-binding site.

### 2.6. NMDAR Fluorescent Competition-Binding Study Using AM-DAN

For these experiments, SH-SY5Y human neuroblastoma cells were used and were prepared as described in detail by our group in previous publications [29,30,31]. The SH-SY5Y cells were added at a density of 1 × 10^5^ cells per 1 mL DMEM in Eppendorf^®^ vials. AM-DAN was used as control (100% fluorescence) and AM-DAN with test derivatives (amantadine and MK-801) were dissolved in DMSO and applied to the cell preparation. The final concentrations (at IC_50_ values for each compound as determined previously [2]) were 15 μM for AM-DAN, 18 μM for amantadine and 0.133 μM for MK-801, respectively. Final DMSO concentrations were kept lower than 2%. A 100 μL solution of 0.55 mM NMDA/glycine solution was then added to selectively stimulate the opening of the NMDAR channel [1,2,27]. After incubation of 5 min at 37 °C the cellular suspension was centrifuged for 2 min using a desk Hermle Z 100 M^®^ Microfuge and the supernatant was discarded. This step was done to remove any unbound AM-DAN. The remaining cells were then carefully resuspended in DMEM buffer (1 mL) and measured spectrofluorometrically (BioTek Synergy fluorescent reader) at the excitation and emission values of AM-DAN (340/488 nm) in DMEM, as there was no spectral shift observed in these experiments. A one-way ANOVA multiple comparison statistical test was conducted on all experimental replicates (n = 3) using GraphPad Prism8^®^.

## 3. Results and Discussion

### 3.1. Energy Minima Studies

**Figure 3:**
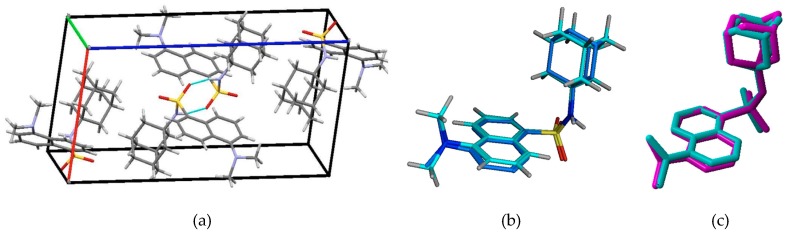
(**a**) The molecular crystal unit-cell packing of AM-DAN viewed along the [010] plain [19]. Hydrogen-bond interactions are shown in cyan. (**b**) Overlap of AM-DAN in its crystalline conformation (cyan) and in its MMFF94 energy minima conformation (blue, RMSD = 0.4107 Å). (**c**) Overlay analysis of the MMFF94 solvated structure (purple) on the MMFF94 gas-phase structure (cyan) (RMSD = 0.016 Å).

Chemical structures in a crystal lattice may differ from those in solution state given the different forces acting from the chemicals’ surroundings [32,33]. Given that AM-DAN would act with the NMDAR outside of the influence of its crystal lattice, we were interested in the conformation of the molecule in terms of an energy minima in which it would remain stable and be appropriate to perform the molecular modelling experiments with high accuracy. The crystallographic data provided the starting geometry of AM-DAN in order to determine the accurate minimum energy structure thereof. The crystal structure, packing arrangement and crystal parameters of AM-DAN have recently been described in Joubert et al., 2019 [19]. Figure 3a illustrates the crystal packing and the important hydrogen-bonds reported [19]. Avogadro 1.2.0 software [21,22] was used to determine the energy minima of AM-DAN free from the crystal field and packing forces using the MMFF94 force field (in gas phase) as described in Section 2.2. In Figure 3b, interestingly, the overlapping images of the optimised AM-DAN and the crystal structure of AM-DAN are almost identical (root-mean-squared-deviation, RMSD = −0.4107 Å), except for a minor rotation of the adamantane portion of the molecule. AM-DAN’s crystal structure is therefore close to the minimum energy conformation and the crystal forces did not influence the conformation thereof significantly. In order to further confirm this statement, a MMFF94 simulation of AM-DAN exposed to explicit water molecules was done using the Water Soak application within MOE [23]. The structures of both the solvated and gas-phase AM-DAN were almost exactly the same (Figure 3c, RMSD = 0.016 Å). This result thus confirms that the crystal forces did not influence the minimum conformation of AM-DAN. 

### 3.2. HOMO and LUMO Calculations

According to the frontier molecular orbital theory the HOMO and the LUMO are the most important orbitals found in a molecule as they may affect biological activity, molecular reactivity, ionisation and electron affinity [34,35,36,37,38,39]. The HOMO is considered the nucleophilic- (electron donating) and the LUMO is the electrophilic (electron accepting) molecular orbital. 

**Figure 4:**
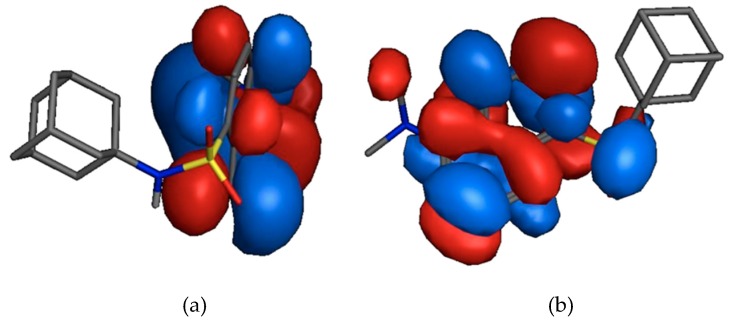
(**a**) A depiction of HOMO on the aryl components of the dansyl moiety and (**b**) LUMO starting from the sulfonamide moiety.

In Figure 4a the HOMO (−8.7265 eV) is localised on the two aryl groups of the dansyl moiety. The LUMO (−0.8856 eV) is located on the sulphonamide moiety and the two aryl groups of the dansyl moiety (Figure 4b). This indicates that the affinity, and potential binding interactions, of the MMFF94 optimised AM-DAN for the PCP binding site of the NMDAR could potentially involve the dansyl moiety and that the adamantane moiety will provide mostly structural bulk, lipophilic function and probable physical blockage of the receptor channel. In addition, the low total energy and large energy gap (7.8409 eV) of HOMO-LUMO suggest that the molecule has good stability and is in its lowest energy conformation.

### 3.3. Fluorescence Studies

**Figure 5:**
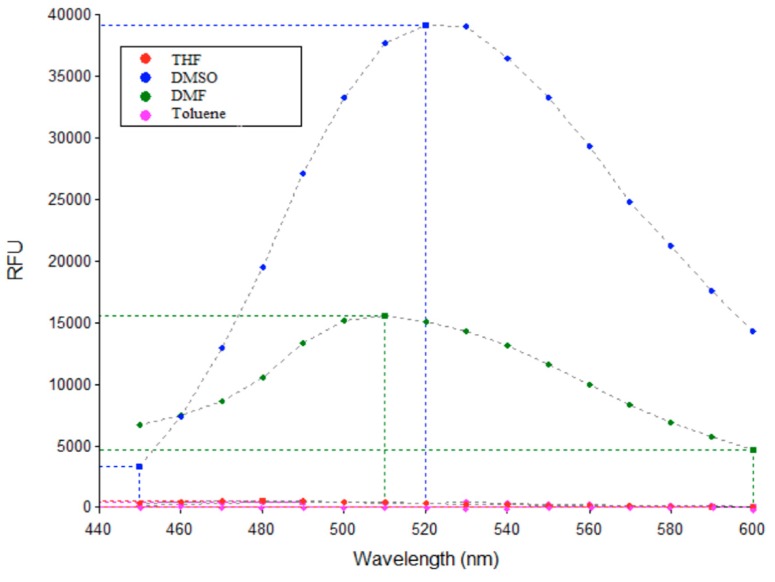
Emission spectra of AM-DAN (10 µM) showing the effect of different aprotic solvents with varying polarities.

The fluorescent properties of AM-DAN were determined by the methods as described in the experimental section. Figure 5 expresses the effect of each aprotic solvent, varying in polarity, on the emission spectrum of AM-DAN. The emission maximum of AM-DAN (final concentration of 10 µM) in all aprotic solvents was observed between 510–520 nm (Figure 5). There was no significant chemical shift observed between the various aprotic solvents. The intensity, however, varied from solvent to solvent with the polar DMSO solvent yielding the greatest increase in intensity (39191 relative fluorescence units, RFU) while both less polar solvents, THF and toluene, yielded almost-negligible fluorescence (510 RFU and 416 RFU, respectively). This indicates that solvent polarity has profound effects on the emission intensity of AM-DAN. The intensity of the fluorescent signal significantly decreased as the solvent polarity decreased (Figure 5). This preliminary study therefore suggests that AM-DAN displays a solvatochromic effect in different aprotic solvent environments of varying polarities, most likely due to an internal charge-transfer phenomenon.

**Figure 6:**
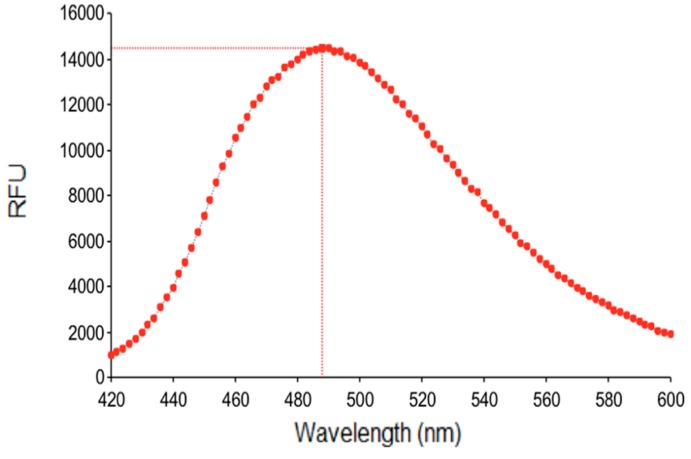
Fluorescent spectrum of AM-DAN (10 µM) in DMEM solution.

In addition, the fluorescence of AM-DAN was also studied in the DMEM medium used in the NMDAR fluorescent competition binding study. Results indicate that measurable fluorescence (RFU = 14,529) was observed for AM-DAN in DMEM, suggesting that it could be appropriate for use in the development of a drug-PCP fluorescent ligand based binding assay (Figure 6). It should however be noted that in the aqueous DMEM solution a fluorescent shift (emission maximum = 488 nm, shift = ±30 nm) was observed when compared to the aprotic organic DMSO and DMF solvents. This may indicate that AM-DAN interacts with surrounding water molecules within the DMEM solution because of the protic nature of water. 

**Figure 7:**
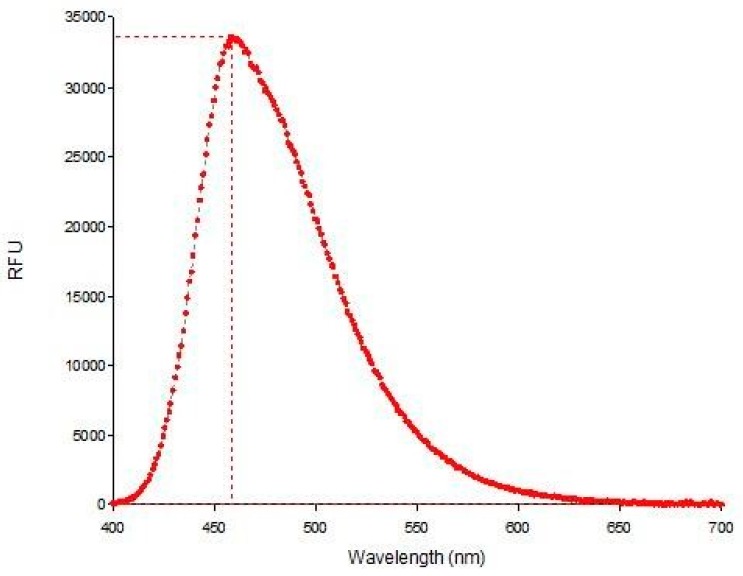
Emission spectrum of solid-state AM-DAN.

The fluorescence of AM-DAN was also determined in its crystalline solid state (Figure 7). A significant blue shift was observed in its emission spectrum when compared to the emission maximum in solution state (emission maximum = 459 nm, organic solvent shift = ±60 nm, DMEM solvent shift = 29 nm). This observation is most likely due to the intermolecular interactions observed in the crystal field (Figure 3a) having altered the chemical and physical properties of the crystalline AM-DAN. This resulted in changes in the photochemical properties as opposed to that of AM-DAN when dissolved in the various solvents, where these intermolecular bonds were no longer present. The fluorescence data collected, when utilised together, provides valuable information that will be used in the effective design of the fluorescent NMDAR ligand-based assay.

### 3.4. Molecular Modelling

#### 3.4.1. Blind Docking

**Figure 8:**
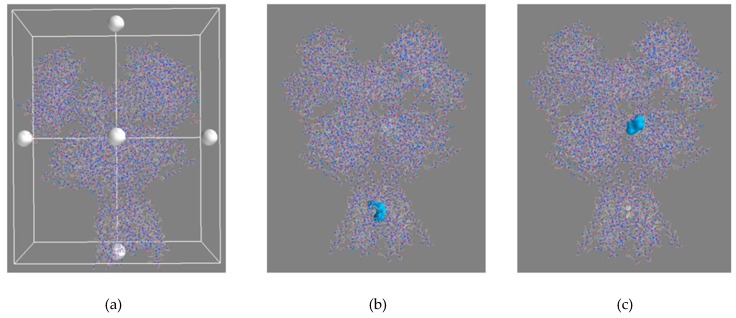
(**a**) The NMDAR (5UOW) with the docking grid placed over the entire protein structure without selecting a binding site. (**b**) Blind docking results showing the overlays of 8 out of the 10 best ranked poses of AM-DAN (blue surface maps) is predicted to binds to the PCP binding site of the NMDAR. (**c**) Results indicating the overlays of the two poses predicted to bind in the vicinity of the glutamate binding site of the NMDAR (blue surface maps).

The PyRx virtual screening tool [24] was used to conduct a blind docking experiment to determine the most probable binding site of AM-DAN within the NMDAR (Figure 8). The blind docking of AM-DAN, using the protein structure of 5UOW, was done by placing the docking grid over the entire NMDAR protein without selecting a binding pocket (Figure 8a). Docking results indicate that 8 out of the 10 best ranked poses of AM-DAN were able to bind within the proximity of the NMDAR PCP binding site (Figure 8b, Table 1) [1,9]. Two poses were predicted to bind in the area of the glutamate binding site [1,9], distinct from the PCP-site (Figure 8c). However, their binding energies were not as promising when compared to the top ranked poses of AM-DAN within the PCP binding site (Table 1). This blind docking experiment therefore indicates that AM-DAN should be able to bind to the PCP site of NMDAR. In order to determine the potential binding interactions of AM-DAN within the PCP binding site, further studies were conducted using MOE software [23] where the PCP-site was selected as the binding pocket (see Section 3.4.2). 

#### 3.4.2. In Silico Structure-Based Docking Studies

**Figure 9:**
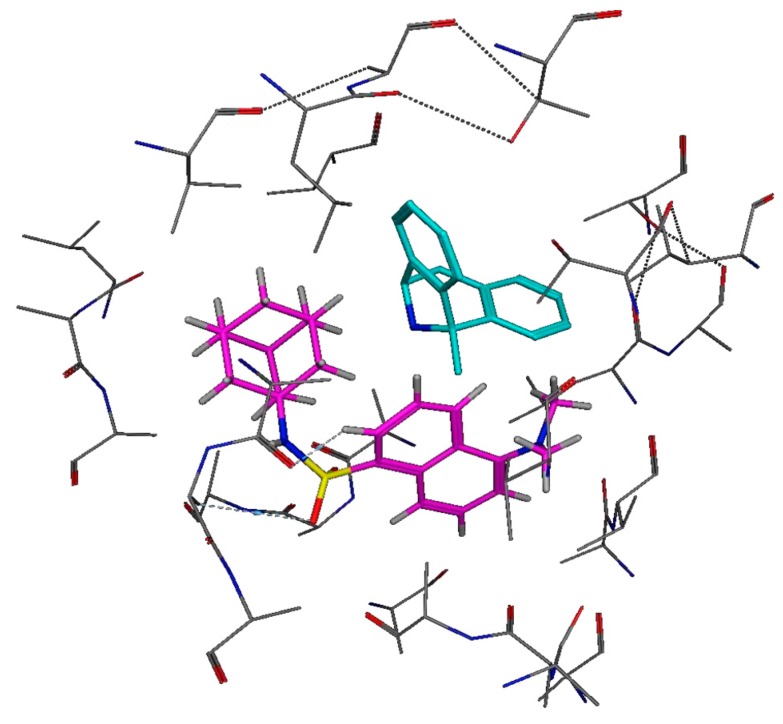
The putative binding modes and orientation of MK-801 (binding affinity = −12.0953 kcal/mol, cyan) and AM-DAN (binding affinity = −10.1640 kcal/mol, magenta) within the PCP-site of the NMDAR.

**Figure 10:**
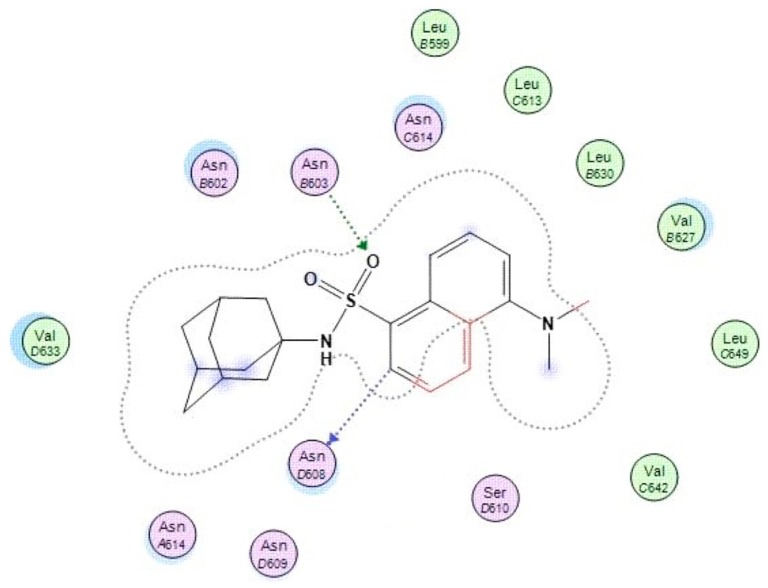
Binding interactions of AM-DAN within the NMDAR PCP-site.

Molecular docking experiments were performed with MOE software [23], using the crystal structure of the NMDAR (Protein Data Bank I.D.: 5UOW), containing MK-801 as ligand (Figure 9) [9]. Molecular modelling studies were conducted in order to explore the interaction profile (Figure 10) and affinity of AM-DAN at the PCP binding site of the NMDAR. The energy minima structure of AM-DAN (MMFF94) was docked according to the protocol described in Section 2.6. AM-DAN was predicted to be able to access, interact and bind to the PCP binding site of the NMDAR within close proximity to MK-801 (Figure 9). Two hydrogen bond interactions were identified: (i) H-bond donor interaction between the heterocyclic ring of the dansyl backbone and ASN608; and an (ii) H-bond acceptor interaction between the S=O moiety of the sulphonamide and ASN603. This result was anticipated, based on the findings of the molecular orbital and theoretical calculations in Section 3.2. The binding affinity of AM-DAN was found to be −10.1640 kcal/mol and correlates well with the binding affinity of MK-801 (−12.0953 kcal/mol, Figure 9). The docking results therefore indicates that AM-DAN could be effective as a fluorescent ligand for NMDAR PCP-site fluorescent displacement studies. 

### 3.5. NMDAR Fluorescent Competition Binding Study Using AM-DAN

**Figure 11:**
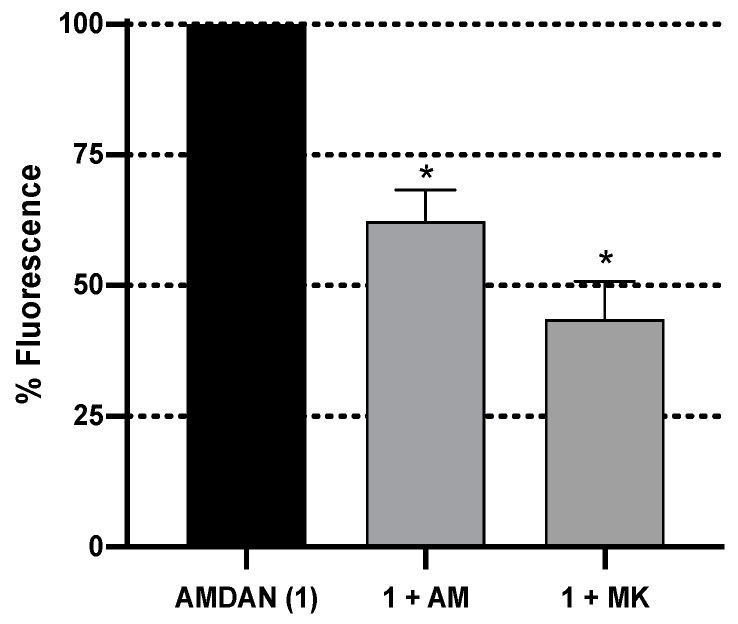
Fluorescent intensities (emission = 488 nm) of NMDA/glycine-mediated competition assay utilising AM-DAN (**1**, 15 μM) and known NMDAR inhibitors, amantadine (AM, 18 μM) and MK-801 (MK, 0.133 μM) in combination with AM-DAN (**1**). Each bar represents mean percentage values ± SEM (*n* = 3), (∗) *p* < 0.05.

A competition assay using SH-SY5Y cells was developed as a proof-of-concept for the potential use of AM-DAN as a PCP-site fluorescent ligand to directly measure drug-PCP binding. The effect of adding NMDAR PCP-site antagonists, amantadine and MK-801 [6,7], in combination with AM-DAN was evaluated (Figure 11). All concentrations used for the test molecules in this assay were at their respective IC_50_ values [2]. The maximal fluorescence in these studies was determined at 488 nm as observed in the fluorescence studies using the DMEM solution. MK-801 decreased the fluorescent intensity of AM-DAN from 100% to 43.56 ± 7.24%. The competition of amantadine with AM-DAN for PCP-site binding also lead to a marked reduction of fluorescent intensity to 62.33 ± 5.96% (Figure 11). 

These results confirm the binding of AM-DAN at the PCP-site and is in-line with the molecular modelling predictions. What is important to note is that both amantadine and MK-801 was able to reduce the fluorescent intensity close to 50%. This is a promising result as it may indicate that AM-DAN is relatively selective and is able to compete for the PCP-site, and potentially does not significantly interact with other cellular structures. In this assay, there was no spectral shift observed upon binding of AM-DAN to the PCP-site as seen for AM-DAN in crystalline state (see Figure 7). As mentioned in Section 3.3, the fluorescent shift observed for the crystalline state of AM-DAN is ascribed to the prominent intermolecular H-bond interactions present in the crystal field. In the molecular modelling studies (see Figure 10), H-bond interactions were also predicted for AM-DAN when bound to the PCP-site and a spectral shift was therefore probable. However, further investigations are necessary to confirm the lack of spectral shift and to fine-tune the assay procedure and parameters. 

## 4. Conclusions

This study was conducted to provide insight into the use of AM-DAN as a fluorescent ligand to measure the affinity of prospective drugs binding at the PCP-site of the NMDAR. Energy minimisation (MMFF94) studies predicted the optimum conformation of AM-DAN and indicated that crystal structure packing forces has little influence on the minimum conformation of the structure. HOMO/LUMO studies showed that the dansyl moiety could be important for binding interactions with the NMDAR PCP-site. In addition, the adamantane group is suggested to mainly provide lipophilic bulk and/or serve as a physical blocker of the channel. Fluorescence studies of AM-DAN in various aprotic solvents and DMEM were conducted successfully, and AM-DAN showed acceptable fluorescent properties. The intensity in fluorescence increased as solvent polarity increased and was almost negligible in the nonpolar aprotic solvents, THF and toluene. Moderate fluorescence was measured in the assay medium—DMEM, suggesting AM-DAN’s appropriateness for use in the competition binding experiment. Docking studies predicted that AM-DAN does in fact bind to the PCP-site of the NMDAR, with binding affinity similar to MK-801. The proof-of-concept competition binding assay using AM-DAN as the fluorescent ligand with known PCP-site antagonists confirmed that the antagonists were able to compete with AM-DAN for this site. However, further biological studies are necessary to explore the potential of AM-DAN as a NMDAR PCP-site fluorescent ligand. Therefore, the next step in this research will be to conduct a series of advanced fluorescent in vitro pharmacological experiments to determine the optimal use of AM-DAN as a novel biological tool in drug discovery that may circumvent the use of hazardous radioligands.

## Figures and Tables

**Table 1:** Predicted binding poses, binding sites and binding energies of the top 10 ranked docking poses of AM-DAN resulting from the blind docking virtual screen.

Pose	Binding Site	Binding Energy (kcal/mol)
1	PCP	−10.3
2	PCP	−9.7
3	PCP	−9.5
4	PCP	−9.2
5	PCP	−9.2
6	PCP	−9.0
7	PCP	−8.9
8	Glutamate	−8.9
9	PCP	−8.8
10	Glutamate	−8.8
